# A Splice Switch in SIGIRR Causes a Defect of IL-37-Dependent Anti-Inflammatory Activity in Cystic Fibrosis Airway Epithelial Cells

**DOI:** 10.3390/ijms23147748

**Published:** 2022-07-13

**Authors:** Keiko Ueno-Shuto, Shunsuke Kamei, Megumi Hayashi, Ayami Fukuyama, Yuji Uchida, Naofumi Tokutomi, Mary Ann Suico, Hirofumi Kai, Tsuyoshi Shuto

**Affiliations:** 1 Laboratory of Pharmacology, Division of Life Science, Faculty of Pharmaceutical Sciences, Sojo University, 4-22-1 Ikeda, Nishi-ku, Kumamoto 860-0082, Japan; kshuto@ph.sojo-u.ac.jp (K.U.-S.); yuchida1@ph.sojo-u.ac.jp (Y.U.); tokutomi@ph.sojo-u.ac.jp (N.T.); 2 Department of Molecular Medicine, Graduate School of Pharmaceutical Sciences, Kumamoto University, 5-1 Oe-Honmachi, Chuo-ku, Kumamoto 862-0973, Japan; skamei@kumamoto-u.ac.jp (S.K.); 212y1010@st.kumamoto-u.ac.jp (M.H.); 191p1050@st.kumamoto-u.ac.jp (A.F.); mann@gpo.kumamoto-u.ac.jp (M.A.S.); hirokai@gpo.kumamoto-u.ac.jp (H.K.); 3 Program for Leading Graduate Schools “HIGO (Health Life Science: Interdisciplinary and Glocal Oriented) Program”, Kumamoto University, 5-1 Oe-Honmachi, Chuo-ku, Kumamoto 862-0973, Japan

**Keywords:** cystic fibrosis (CF), single immunoglobulin interleukin-1 receptor (IL-1R)-related molecule (SIGIRR), IL-37, anti-inflammation, splicing switch, airway epithelial cells

## Abstract

Cystic fibrosis (CF) is a hereditary disease typically characterized by infection-associated chronic lung inflammation. The persistent activation of toll-like receptor (TLR) signals is considered one of the mechanisms for the CF hyperinflammatory phenotype; however, how negative regulatory signals of TLRs associate with CF inflammation is still elusive. Here, we showed that the cell surface expression of a single immunoglobulin interleukin-1 receptor (IL-1R)-related molecule (SIGIRR), a membrane protein essential for suppressing TLRs- and IL-1R-dependent signals, was remarkably decreased in CF airway epithelial cells compared to non-CF cells. Notably, CF airway epithelial cells specifically and highly expressed a unique, alternative splice isoform of the SIGIRR that lacks exon 8 (Δ8-SIGIRR), which results in the production of a *C*-terminal truncated form of the SIGIRR. Δ8-SIGIRR was expressed intracellularly, and its over-expression abolished the cell surface expression and function of the full-length SIGIRR (WT-SIGIRR), indicating its dominant-negative effect leading to the deficiency of anti-inflammatory activity in CF cells. Consistently, IL-37, a ligand for the SIGIRR, failed to suppress viral dsRNA analogue poly(I:C)-dependent JNK activation and IL-8 production, confirming the reduction in the functional WT-SIGIRR expression in the CF cells. Together, our studies reveal that SIGIRR-dependent anti-inflammatory activity is defective in CF airway epithelial cells due to the unique splicing switch of the SIGIRR gene and provides the first evidence of IL-37-SIGIRR signaling as a target of CF airway inflammation.

## 1. Introduction

Cystic fibrosis (CF) is the most common lethal inherited disorder in Caucasians that is mainly characterized by recurrent bacterial and viral infections leading to neutrophil-dominated chronic lung inflammation induced by excessive amounts of IL-8 production, a major regulator of CF airway inflammation [1,2]. The infection-associated chronic lung inflammation is caused by defects in the cAMP-dependent Cl^-^ channel CF transmembrane conductance regulator (CFTR), a causative protein of hereditary CF [3]. In the CF lung, CFTR dysfunction causes an imbalance between fluid absorption and secretion, resulting in airway mucus hyperproduction, dysregulated airway clearance, and enhanced inflammation to microbial infection [4,5]. Some argue that accumulated misfolded CFTR proteins in the endoplasmic reticulum (ER) lead to inflammatory responses via an unfolded protein response (UPR) [6]; others have focused on the possible intrinsic effects of a mutant CFTR, not only on the airway epithelial cells but also on the immune system for CF hyperinflammation [7,8]. Although there are still big debates on the cause of hyperinflammation in CF, the dysregulated expression of many of the genes in CF patients- and CF animal model-derived airway epithelial cells has been proposed to be one of the well-discussed molecular mechanisms underlying hyperresponsiveness to the microbial ligands [6]. A group of such genes associated with infection and inflammation in airway epithelial cells is the toll-like receptors (TLRs), innate immune receptors that sense pathogen-associated molecular patterns (PAMPs) and induce inflammatory responses [9,10]. Many studies have demonstrated the increased activation of TLR2, TLR4, and TLR5 in CF airway epithelial cells and macrophages, which contributes to a hyperinflammatory response toward bacterial peptidoglycan (PGN), lipopeptides, lipopolysaccharide (LPS), and flagellin from *Staphylococcus aureus* and *Pseudomonas aeruginosa* that are important pathogens in pulmonary infections in CF [11,12,13,14,15,16,17]. Additionally, infections with respiratory viruses, including rhinovirus and influenza A and B, are strongly associated with pulmonary exacerbations due to a deficient anti-viral activity in the CF airway surface liquid (ASL), leading to increased viral replication and cytokine production via TLR3 [18,19,20].

The dysregulation of the pathways that negatively regulate TLR signaling, including T1/ST2, the single immunoglobulin interleukin-1 receptor (IL-1R)-related molecule (SIGIRR), a splicing variant of the myeloid differentiation factor 88 (MyD88s), a suppressor of cytokine signaling 1 (SOCS1), Triad3A, CYLD, and IRAK-M, were well-described in many inflammatory diseases [21,22,23]; however, little is known about if and how these negative regulatory signals of TLRs contribute to CF inflammation.

The SIGIRR (also known as the interleukin-1 receptor 8, IL-1R8, or TIR8) is a member of the IL-1 receptor (ILR) family, with distinct structural and functional characteristics acting as a negative regulator of multiple ILR and TLR downstream signaling pathways and inflammation [24]. Its extracellular domain was shown to block the dimerization of ILR proteins, while its intracellular TIR domain was shown to bind the TIR-containing adaptor proteins, such as MyD88, to sequester downstream signaling molecules [25,26,27,28]. Notably, although the SIGIRR was originally considered an orphan receptor, which lacks a specific ligand, recent studies have identified an anti-inflammatory cytokine IL-37 as an endogenous ligand for the SIGIRR. After IL-37 binds to the alpha chain of the IL-18 receptor (IL-18Rα), the SIGIRR is recruited to the complex to dampen pro-inflammatory and transduce anti-inflammatory signals [29]. Among the above-mentioned TLR negative regulators, the SIGIRR is highly expressed in various epithelial tissues, especially in the lung [25,30]. Therefore, we speculate that SIGIRRs may maintain an activation threshold of ILR and TLR signaling in the lung, and its expression and/or function may be dysregulated in CF airway epithelial cells.

In this study, we first showed a marked downregulation of the cell surface of the SIGIRR as a common feature in the CFTR-defective CF airway epithelial primary cells and cell lines, which hinted at the contributory roles of SIGIRRs in the regulation of CF airway inflammation. Notably, the CF cells highly expressed a unique alternative splice isoform of the SIGIRR whose exon 8 was deleted (Δ8-SIGIRR). It was mainly retained in the ER and interfered with the cell surface expression of the full-length wild-type SIGIRR (WT-SIGIRR), suggesting its dominant-negative function. Accordingly, treatment with a precursor or truncated form of IL-37b, the well-identified endogenous SIGIRR ligand, failed to suppress viral dsRNA analogue poly(I:C)-dependent IL-8 production in the CF cells but not in the non-CF cells. Finally, the ectopic WT-SIGIRR expression restored the IL-37b-dependent anti-inflammatory effect in the CF cells, implicating that the unique splicing switch of the SIGIRR gene would be a possible therapeutic target for virus-associated lung inflammation in CF.

## 2. Results

### 2.1. The Expression of Cell Surface SIGIRR Is Downregulated in CF Cells

**Figure 1:**
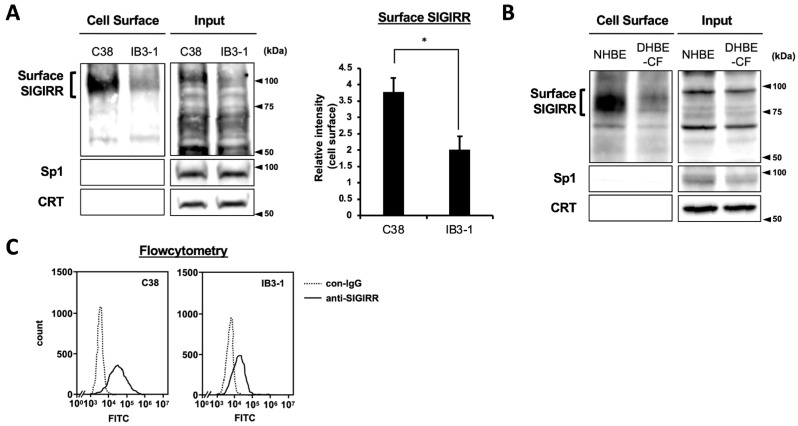
The expression of the cell surface of the SIGIRR is downregulated in CF cells. (**A**,**B**) Cell surface expression of the SIGIRR in (**A**) C38 and IB3-1, (**B**) NHBE and DHBE-CF (non-CF vs. CF) airway epithelial cells were assessed by biotinylation assay, followed by immunoblotting using anti-SIGIRR, calreticulin (CRT) and Sp1 antibodies. Protein bands in (**A**) were scanned, and relative band intensities were normalized to the β-actin band. The graphs represent the average relative band intensity with S.D. from four independent experiments. * *p* < 0.05; Student’s *t*-test (*n* = 3). (**C**) Flow cytometry for the SIGIRR was performed using C38 and IB3-1 cells. Surface expressions of the SIGIRR were indicated by a fluorescence shift compared to the isotype control antibody (dotted line).

To compare the expression levels of the cell surface of the SIGIRR between non-CF and CF airway epithelial cells, we carried out a biotinylation assay using non-CF and CF cell pairs (C38 vs. IB3-1, NHBE vs. DHBE-CF). A heavily glycosylated form of the SIGIRR of around 100 kDa, which is primarily expressed on the cell surface, was drastically diminished in the IB3-1 cells compared with the C38 cells (Figure 1A). Decreased levels of the SIGIRR were also observed in the DHBE-CF cells compared with the NHBE cells (Figure 1B). Consistently, flow cytometric analysis revealed a lower expression of the cell surface of the SIGIRR in the IB3-1 cells compared with the C38 cells (Figure 1C).

### 2.2. Enhanced Expression of Alternative Isoform of SIGIRR in CF Cells

**Figure 2:**
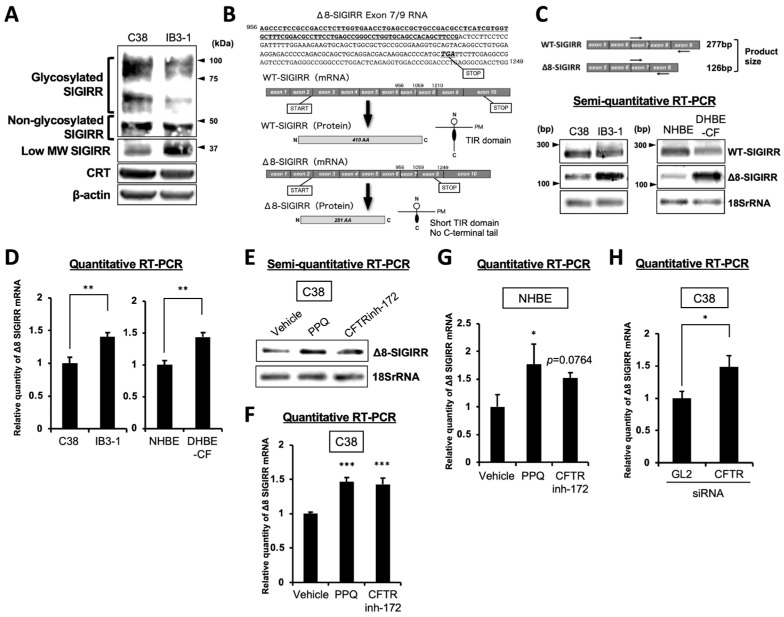
Enhanced expression of the alternative isoform of Δ8-SIGIRR in CF cells. (**A**) The SIGIRR protein expression in C38 and IB3-1 cells was analyzed by immunoblotting. (**B**) The sequence of human Δ8-SIGIRR from exon 7 to 9 and schematic representation of the human WT-SIGIRR and Δ8-SIGIRR. Bold, underlined text indicates the exon 7 region, and italic lettering shows the premature stop codon caused by the frameshift. (**C**) The mRNA levels of WT- and Δ8-SIGIRR in non-CF and CF cells were assessed by semi-quantitative RT-PCR using specific primers for exons 7 and 9. The data were normalized to 18SrRNA mRNA levels. (**D**) The mRNA levels of Δ8-SIGIRR in non-CF and CF cells were determined by quantitative RT-PCR using primers targeting the exon 7-exon 9 junction of Δ8-SIGIRR. The data were normalized to 18S rRNA mRNA levels. ** *p* < 0.01 versus non-CF cells; Student’s *t*-test (*n* = 3). (**E**–**G**) The non-CF cells were treated with CFTR inhibitors (15 μM PPQ and 30 μM CFTRinh-172) for 24 h. The mRNA levels of Δ8-SIGIRR were assessed by semi-quantitative (**E**) or quantitative RT-PCR (**F**,**G**). The data were normalized to 18SrRNA mRNA levels. * *p* < 0.05, *** *p* < 0.001 versus the vehicle-treated cells; ANOVA with Dunnett’s test (*n* = 3). (**H**) C38 cells were transfected with 20 nM of siRNA against GL2 (control) or the CFTR for 48 h, and then the mRNA levels of Δ8-SIGIRR were assessed by quantitative RT-PCR. The data were normalized to 18SrRNA mRNA levels. * *p* < 0.05 versus the GL2-siRNA transfected cells; Student’s *t*-test (*n* = 3). The values shown represent the mean ± S.D.

To determine the form of SIGIRRs expressed in whole-cell lysates, immunoblotting was performed in the C38 and IB3-1 cells. The data showed that the expression of the glycosylated SIGIRR was decreased in the IB3-1 cells but not in the non-glycosylated form (Figure 2A). On the other hand, the low molecular-weight of the SIGIRR expression at 37 kDa was enhanced in the IB3-1 cells, implying possible alternative splicing (Figure 2A).

Zhao et al. previously demonstrated that the exon 8-deleted SIGIRR (Δ8-SIGIRR) cannot be modified by complex glycans and cannot be localized to the plasma membrane due to its ER retention [31]. Exon 8 deletion creates a stop codon that leads to a lack of the TIR domain at the C-terminus, which is essential for the anti-inflammatory function of the SIGIRR (Figure 2B). Furthermore, Δ8-SIGIRR functions as a dominant-negative mutant of the wild-type SIGIRR (WT-SIGIRR) because Δ8-SIGIRR sequesters the WT-SIGIRR in the ER, preventing its glycan modification and localization to the plasma membrane [31]. To assess the Δ8-SIGIRR expression in non-CF and CF airway epithelial cells, we amplified the segment between exons 7 and 9 by semi-quantitative RT-PCR. Consistent with the increased protein expression of the low molecular weight of the SIGIRR, the mRNA levels of Δ8-SIGIRR were enhanced in the CF cells (IB3-1 and DHBE-CF) compared with the non-CF cells (C38 and NHBE) (Figure 2C). The results were also confirmed by a quantitative RT-PCR (Figure 2D), suggesting that the alternative isoform, Δ8-SIGIRR, was up-regulated in the CF airway epithelial cells.

These observations prompted us to investigate whether the inhibition of the CFTR channel function or CFTR gene knockdown led to an Δ8-SIGIRR up-regulation in the non-CF cells. The CFTR inhibitors’, quinoxalinedione (PPQ)-102 and CFTRinh-172, treatment enhanced the Δ8-SIGIRR expression in the C38 cells (Figure 2E,F) and NHBE cells (Figure 2G). Furthermore, the CFTR gene knockdown also upregulated Δ8-SIGIRR in the C38 cells (Figure 2H), implying that a CF-like intracellular environment, due to the lack of a functional CFTR, might facilitate alternative splicing to produce Δ8-SIGIRR.

### 2.3. Δ8-SIGIRR Impedes Cell Surface Expression of WT-SIGIRR

**Figure 3:**
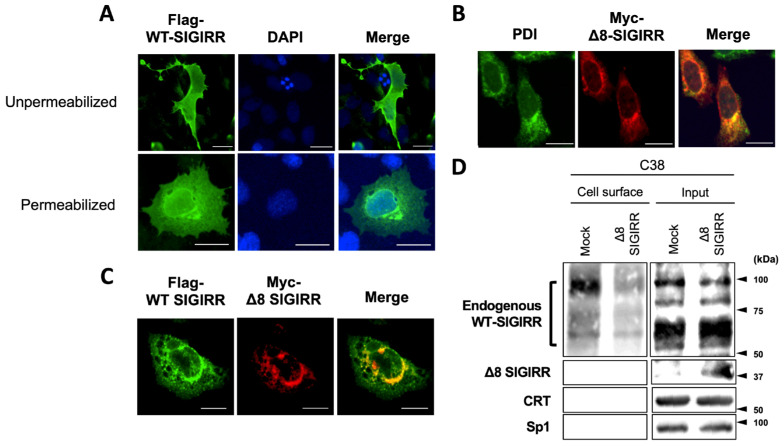
Δ8-SIGIRR impedes the cell surface expression of the WT-SIGIRR in C38 cells. (**A**) Immunofluorescence staining of exogenously expressed the Flag-WT-SIGIRR in C38 under unpermeabilized and permeabilized conditions. Green indicates Flag-directed Alexa Fluor^®^ 488 fluorescence. Blue indicates the DAPI nuclear counter stain. (**B**) Immunofluorescence staining of exogenously expressed Myc-Δ8-SIGIRR in C38 under permeabilized conditions. Green indicates the ER marker protein disulfide isomerase (PDI)-directed Alexa Fluor^®^ 488, and red indicates Myc-directed Alexa Fluor^®^ 594 fluorescence. (**C**) Immunofluorescence staining of exogenously expressed the Flag-WT-SIGIRR and Myc-Δ8-SIGIRR in C38 under permeabilized conditions. Green indicates FLAG, and red indicates Myc-directed Alexa Fluor^®^ 488 and 594 fluorescence, respectively. Scale bar = 20 μm. (**D**) C38 transfected with pcDNA, or Δ8-SIGIRR was assessed by biotinylation assay, followed by immunoblotting with anti-SIGIRR, -CRT, and -Sp1 antibodies.

To visualize the intracellular localization and dominant-negative function of Δ8-SIGIRR, immunofluorescence staining was performed in the ectopic WT- and/or Δ8-SIGIRR-expressed C38 cells. Under both unpermeabilized and permeabilized conditions, the Flag-tagged WT-SIGIRR alone was expressed throughout the cells that included a peripheral edge, implying its cell surface expression (Figure 3A, Supplemental Figure S1). In contrast, Myc-tagged Δ8-SIGIRR alone accumulated at the perinuclear region and merged with the ER marker PDI (Figure 3B). After the co-transfection of the two types of SIGIRRs, we found that the Flag-tagged WT-SIGIRR lost the peripheral edge staining and mostly co-localized with Myc-tagged Δ8-SIGIRR intracellularly, suggesting the ability of Δ8-SIGIRR to impede the cell surface expression of the WT-SIGIRR (Figure 3C). In support of this, the ectopic Δ8-SIGIRR expression attenuated the surface expression of the complex-glycosylated WT-SIGIRR in the C38 cells (Figure 3D). Collectively, these results indicate that Δ8-SIGIRR plays a dominant-negative role in suppressing the complex-glycosylation and cell surface expression of the WT-SIGIRR in airway epithelial cells.

### 2.4. IL-37b, a Ligand for SIGIRR, Fails to Suppress Poly(I:C)-Dependent IL-8 Production and JNK Phosphorylation in CF Cells

IL-37 is a member of the IL-1 family that is ubiquitously expressed in a variety of hematopoietic cells, as well as in epithelial cells, including the lung. Among the five isoforms termed IL-37a–e, IL-37b is the largest and best-characterized isoform that is released from the cells as a precursor form. The Il-37b precursor is processed into the *N*-terminal 45 amino acids in a truncated form (Val46) in the extracellular space [30]. Unlike other IL-1 family members, IL-37b broadly suppresses innate and adaptive immunity, and its anti-inflammatory property is in part mediated by the tripartite complex of the IL-37b-IL-18Rα-SIGIRR on the cell surface [30,31]. Because the C-terminus of the SIGIRR has the ability to sequester the inflammatory signaling molecules after its recruitment to the IL-37b-IL18Rα complex, the regulation of the SIGIRR is largely relevant to the anti-inflammatory effects of IL-37b.

**Figure 4:**
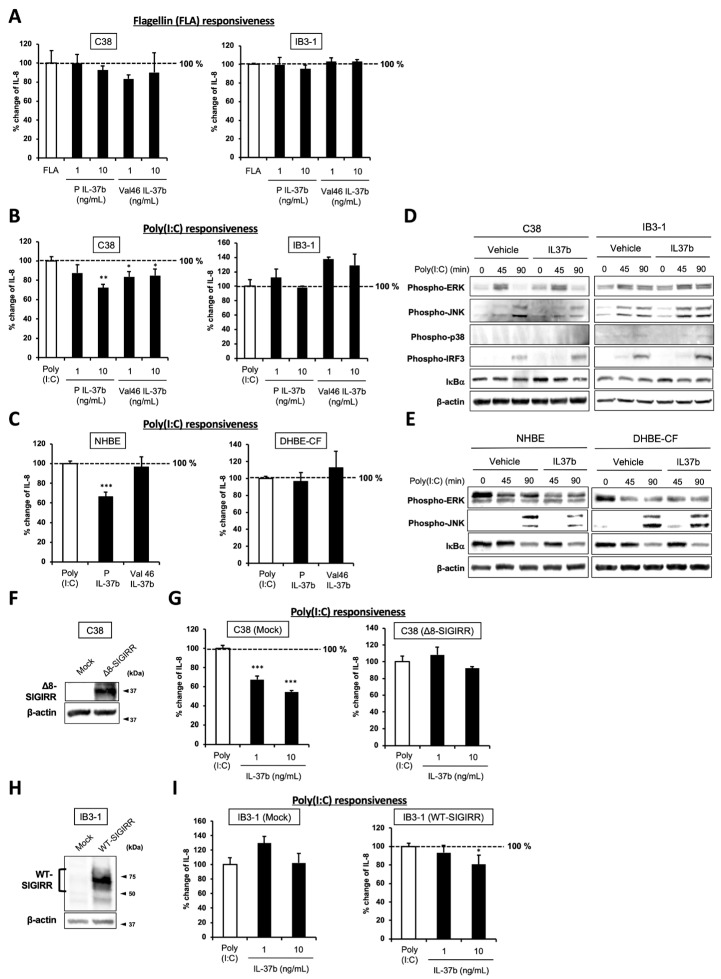
Suppression of IL-8 production by IL-37b is dependent on the cell surface-expressed WT-SIGIRR, leading to the attenuation of JNK phosphorylation. (**A**,**B**) C38 and IB3-1 cells were stimulated with 1 μg/mL flagellin or 20 μg/mL poly(I:C) 2 h after treatment with the indicated concentrations of a precursor or Val46 IL-37b. After 24 h, released IL-8 in the condition media was measured by ELISA. (**C**) NHBE and DHBE-CF cells were stimulated with 0.5 μg/mL poly(I:C) 2 h after treatment with 10 ng/mL of a precursor or Val46 IL-37b. After 24 h, released IL-8 in the condition media was measured by ELISA. (**D**) C38 and IB3-1 cells and (**E**) NHBE and DHBE-CF cells were stimulated with poly(I:C) (**D**, 20 μg/mL; **E**, 1 μg/mL) 2 h after treatment with a 10 ng/mL precursor of IL-37b. After the indicated period of the poly(I:C) treatment, the total cell lysates were subjected to immunoblotting using antibodies against the phospho-active form of MAPKs, IRF3, and IκBα. (**F**,**G**) pcDNA or Δ8-SIGIRR-transfected C38 cells and (H and I) pcDNA or the WT-SIGIRR-transfected IB3-1 cells were stimulated with 20 μg/mL poly(I:C) 2 h after treatment with the indicated concentrations of the precursor of IL-37b. After 24 h, released IL-8 in the condition media was measured by ELISA. Exogenously expressed Δ8 and the WT-SIGIRR were assessed by immunoblotting with the anti-SIGIRR. For the immunoblotting experiments (**D**–**F**,**H**), β-actin was used as a loading control. (**A**–**C**,**G**,**I**) Results represent the mean ± S.D. * *p* < 0.05, ** *p* < 0.01, *** *p* < 0.001 versus poly(I:C)-treated cells; ANOVA with Dunnett’s test (*n* = 3).

To investigate whether IL-37b exhibits anti-inflammatory activity in the TLR ligand-stimulated non-CF and CF airway epithelial cells, we pretreated the C38 and IB3-1 cells with a precursor, or Val46 IL-37b before their exposure to flagellin (FLA), a ligand for TLR5, or poly(I:C), a synthetic ligand of TLR3, and then the cytokine production was measured by ELISA. The FLA-induced IL-8 production was slightly suppressed by the Val46 IL-37b treatment, but the difference was not significant in the C38 cells (Figure 4A). Under the same condition, there was no change in the IL-8 production in the IB3-1 cells. In the case of the poly(I:C) exposure, both the precursor and Val46 IL37b treatment significantly suppressed IL-8 production in the C38 cells, whereas they failed to suppress IL-8 production in IB3-1 cells (Figure 4B). Furthermore, only the precursor form attenuated poly(I:C)-induced IL-8 production in NHBE cells but not in the DHBE-CF cells (Figure 4C), indicating that the precursor form is more effective than the truncated form in this system.

To further explore the mechanisms responsible for the precursor IL-37b-dependent IL-8 suppression, we examined the activation of signaling molecules downstream of TLR3 with or without IL-37b treatments by immunoblotting. As shown in Figure 4D, the IL-37b treatment attenuated only a JNK phosphorylation in C38 but not in the IB3-1 cells. Similar results were observed in the IL-37b-treated NHBE and DHBE-CF cells (Figure 4E). Previous reports demonstrated that the AP-1 downstream of JNK is essential for IL-8 induction in airway epithelial cells [1,32,33,34,35], suggesting JNK suppression as a possible target of IL-37b-dependent anti-inflammatory property.

### 2.5. Cell Surface-Expressed WT-SIGIRR Contributes to IL-37b-Dependent IL-8 Suppression

To determine whether suppression of IL-8 production by IL-37b is dependent on the cell surface-expressed WT-SIGIRR, the C38 and IB3-1 cells were transfected with Δ8-SIGIRR and the WT-SIGIRR, respectively, and the cells were pretreated with the indicated concentrations of IL-37b followed by poly(I:C) exposure. The IL-37b-dependent IL-8 suppression was completely abolished by the transfection of Δ8-SIGIRR (Figure 4F,G). This result is consistent with the dramatic downregulation of the cell surface expression of the WT-SIGIRR by the Δ8-SIGIRR expression in the C38 cells (Figure 3D). In contrast, the WT-SIGIRR overexpression in the IB3-1 cells restored the WT-SIGIRR and exhibited an IL-37b-dependent IL-8 suppression in the IB3-1 cells (Figure 4H,I). Together, our results indicate that the cell surface expressing the functional WT-SIGIRR contributes to an IL-37b-dependent IL-8 suppression, presumably leading to the attenuation of JNK phosphorylation.

## 3. Discussion

**Figure 5:**
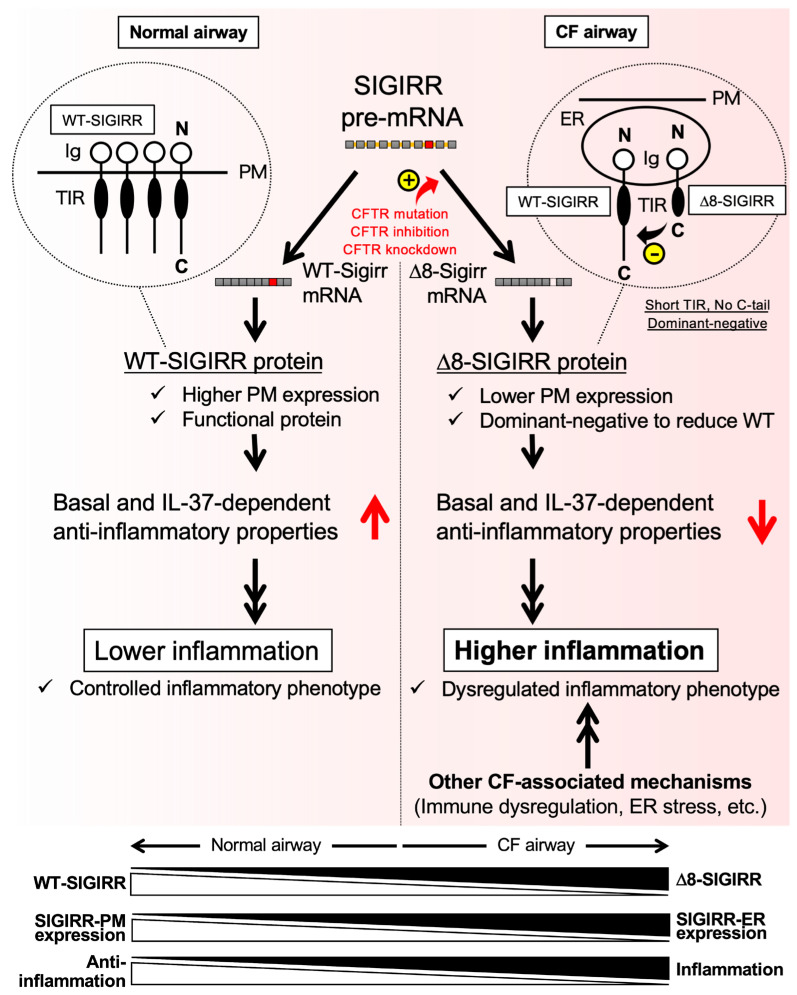
Putative mechanisms of IL-37-SIGIRR-dependent anti-inflammatory activity defected in CF airway epithelial cells due to the unique splicing switch of the SIGIRR gene. In normal and non-CF airway epithelial cells (left panel), the WT-SIGIRR mRNA-derived WT-SIGIRR protein contributes to maintaining the basal and IL-37-dependent anti-inflammatory properties, resulting in the controlled inflammatory phenotype. In CF airway epithelial cells (right panel), a splice switch from the WT-SIGIRR to Δ8-SIGIRR, an alternative splice isoform of the SIGIRR, which lacks exon 8 (Δ8-SIGIRR), contributes to the production of the *C*-terminal truncated form of the SIGIRR protein. The Δ8-SIGIRR protein exhibits a lower plasma membrane (PM) expression and has a dominant-negative effect with the WT-SIGIRR-interfering activity; thus, the splice switch to Δ8-SIGIRR causes a reduction in the basal and IL-37-dependent anti-inflammatory properties in CF airway epithelial cells, resulting in the hyperinflammatory phenotype. Other CF-associated mechanisms, such as immune dysregulation and ER stress, may also contribute to the pathogenesis of CF hyperinflammation. Overall, the balance of the WT-SIGIRR and Δ8-SIGIRR affects the PM expression levels of the WT-SIGIRR, which control the anti-inflammatory phenotype in airway epithelial cells.

Although the average life span for CF patients is lengthened due to breakthrough drugs developed over the past few years [36,37,38,39], novel anti-inflammatory therapeutic approaches are still promising to control neutrophil-dominated chronic lung inflammation in CF. In this study, we identified the defective surface expression of the WT-SIGIRR, possibly through a mechanism by accelerated conversion to the exon 8-deleted alternative isoform Δ8-SIGIRR that functions as a dominant-negative against the WT-SIGIRR in CF airway epithelial cells (Figure 5). Because the decreased surface WT-SIGIRR levels, in accordance with the defective anti-inflammatory IL-37 signal, could contribute to CF-associated hyperinflammation in the airway epithelial cells, these findings uniquely link splice switching in the SIGIRR and CF-associated airway inflammation. In general, hyperinflammation, especially under airway clearance-defective conditions observed in the CF airways, is an exacerbating factor [12,40,41,42,43,44,45,46,47,48]. Thus, the identification of the molecules and pathways underlying CF-associated hyperinflammation is urgently needed. However, in another aspect, strong inflammation may be required to exert proper host defenses as a main component of innate immune responses, especially under the infection stage of CF patients [49]. In any case, our findings on the importance of the IL-37-SIGIRR axis and splice switch of the SIGIRR could provide a novel target against CF-associated hyperinflammatory or less innate immune responses.

The molecular mechanisms underlying the CF-specific alternative splicing of the SIGIRR has yet to be fully defined, but our recent study has provided a first unique example of a splicing switch in a CF-specific manner [50]. The report showed that another alternative splicing product, ΔC-ZIP2, an alternative splice isoform that lacks the *C*-terminal domain of the zinc importer, ZIP2, is produced in CF airway epithelial cells. Similar to our present study, the ΔC-ZIP2 alternative splicing is induced under a CFTR-defective condition, and the CFTR complementation in the CF cells restores the WT-ZIP2 [50]. In addition, the study further showed the epithelial sodium channel (ENaC), another ion transporter that is commonly hyperactivated in CF cells, as a crucial ΔC-ZIP2 splicing-inducing factor. Because the inverse relationship between the CFTR and ENaC functions has been widely accepted in CF research fields [51], future mechanistic studies need to be performed with cellular and animal models of ENaC-hyperactivation [50,52].

The identification of the anti-inflammatory IL-37b-SIGIRR axis as one of the defective signals in CF cells provides a novel insight into understanding CF inflammation. Consistently, a number of inflammatory models have revealed the protective effects of IL-37, either in IL37-transgenic (Tg) mice or in mice treated with recombinant IL-37. For example, IL37-Tg mice subjected to dextran sulfate sodium (DSS)-induced colitis, as well as spinal cord injury, were protected against the development of disease phenotypes [53,54]. Moreover, exogenous treatment with recombinant IL-37 exerted anti-inflammatory properties in collagen-induced arthritis (CIA) models, as well as in T cells from patients with rheumatoid arthritis [55]. Notably, lower levels of IL-37 were determined in patients with not only typical inflammatory diseases, such as psoriasis, rheumatoid arthritis, and Behcet’s disease, but also with some airway-associated inflammatory diseases, including nasal polyps, allergic rhinitis, asthma, and non-small cell lung cancer [33]. Overall, our study encourages IL-37 activation (modulation) as a potential novel therapeutic tool against CF.

As a technical limitation, the present study did not rule out whether the defect in the IL-37b-SIGIRR axis is a general feature in CF airway epithelial cells. To date, there is a number of isogenic CF airway cell pairs that show the intrinsic alteration of inflammatory phenotypes [11,12,13,14,17], as is the case with the C38 and IB3-1 cell lines in this study. However, the CF-specific phenotypic difference seems to depend on the airway epithelial pairs. For instance, Hybiske et al. adenovirally transfected human CF nasal cell lines (CF15) with the wild-type- and ΔF508-CFTR and found no intrinsic differences in terms of basal and infection-triggered inflammation [56]. Although our data were confirmed with human primary airway epithelial pairs, further experiments with other isogenic CF pairs must be performed to understand the general contributory role of the IL-37b-SIGIRR axis in the regulation of CF inflammation.

CF inflammatory research has been mainly focused on airway epithelial cells for many years. However, recent studies have moved towards new trends showing the intrinsic effects of the mutant CFTR on the immune system, such as neutrophils, macrophages, lymphocytes, and dendritic cells [7]. Notably, we have previously shown that the SIGIRR is also expressed in neutrophilic and monocytic cells [57], and others also revealed an expression of the SIGIRR and IL-37 in several immune cells [58]. In this connection, the CFTR-SIGIRR-IL-37 axis could also be conserved in the immune system and may also contribute to the pathogenesis of CF airway inflammation.

Finally, the present study firstly shows the importance of Δ8-SIGIRR in the inflammatory regulation of the CF airway, although the pathological relevance of Δ8-SIGIRR has already been shown in one study. Zhao et al. revealed that human colorectal cancer has a higher expression of Δ8-SIGIRR, which results in the suppression of the plasma membrane of the WT-SIGIRR expression [31], as is similar to the present study. Because tumor suppression is one of the key functions of the SIGIRR, Δ8-SIGIRR could be used as a prognostic marker of colorectal cancer. In the clinical aspect, whether CF patients’ condition and prognosis can also be estimated with the Δ8-SIGIRR expression is an issue that needs to be further clarified. In addition, understanding the pathological relevance of Δ8-SIGIRR in other diseases opens up a new paradigm in the development of novel SIGIRR-targeting drugs. Together, our data unveil a connection between the Δ8-SIGIRR expression and hyperinflammatory phenotype at the cellular level of CF airways and provide the novel concept that control of the SIGIRR splice switch, as well as IL-37 activation, could be previously undetermined targets for CF anti-inflammatory therapeutics.

## 4. Materials and Methods

### 4.1. Cell Culture and Reagents

The IB3-1 (bronchial epithelial cell line from a CF patient with genotype ΔF508/W1282X CFTR mutations) and C38 (a “corrected” CF cell line derived from IB3-1 after stable transfection with the CFTR) were obtained from the American Type Culture Collection (ATCC) and grown on precoated dishes (100 μg/mL BSA, 30 μg/mL pig collagen type I, 10 μg/mL human fibronectin) at 37 °C in a humidified 5% CO_2_ atmosphere in gentamicin-free LHC-8 (Biofluids, Rockville, MD, USA) supplemented with 5% FBS (Thermo Scientific Inc., Waltham, MA, USA), 100 U/mL of penicillin and 100 mg/mL of streptomycin (Nacalai Tesque, Inc., Kyoto, Japan). Normal human bronchial epithelial cells (NHBE) and cystic fibrosis human bronchial epithelial cells (DHBE-CF) were purchased from Lonza (Walkersville, MD, USA) and maintained as described previously [59]. CFTR expression levels at the mRNA and protein were confirmed by quantitative RT-PCR and immunoblot analysis, respectively (Supplemental Figure S2). All cells were cultured in a humidified incubator at 37 °C with 5% CO_2_. The low molecular weight (LMW) poly(I:C) variant (0.2–1 kb) and ultrapure flagellin from *Pseudomonas aerginosa* were purchased from InvivoGen (San Diego, CA, USA). The PPQ-102 and CFTR inhibitor-172 were purchased from Cayman Chemical (Ann Arbor, MI, USA). The recombinant precursor and Val46 IL37b were purchased from R&D systems (Minneapolis, MN, USA).

### 4.2. RNA Isolation, cDNA Synthesis, and Quantitative and Semi-Quantitative RT-PCR Analysis

Total RNA extraction from non-CF and CF cell lines or primary cells was performed using NucleoSpin RNA Plus (TaKaRa, Kusatsu, Japan) according to the manufacturer’s protocols. 0.25 μg of total RNA was then used for the reverse transcription reaction using a PrimeScript^®^ RT master mix (TaKaRa). Quantitative RT-PCR was performed in PikoReal^TM^ real-time PCR detection systems (Thermo Scientific Inc.), and the gene expression was examined by TB green^®^ Premix Ex TaqTM II (TaKaRa). The relative quantity of the target gene mRNA was normalized using human 18SrRNA as the internal control and expressed as the relative quantity of the target gene mRNA (-fold induction). PCR amplification was performed in triplicate, and the reaction protocol included pre-incubation at 95 °C to activate Ex Taq HS for 30 s; amplification of 40 cycles was set for 15 s at 95 °C, and annealing was set for 60 s at 60 °C. Semi-quantitative RT-PCR was conducted using a PrimeSTAR Max DNA Polymerase (TaKaRa) according to the manufacturer’s protocols. PCR was performed for 30 cycles at 98 °C for 10 s, 55 °C for 5 s, 72 °C for 5 s (for the SIGIRR), or 15 cycles at 98 °C for 10 s, 55 °C for 5 s, 72 °C for 5 s (for 18SrRNA). The sequences of the primers used for quantitative and semi-quantitative RT-PCR are shown in Table 1.

### 4.3. Immunofluorescence (IF) Staining

Intracellular localization of the exogenous WT or Δ8 human SIGIRR was examined by IF staining. The cells, plated on glass-bottom dishes, were washed twice with a phosphate-buffered saline (PBS) and fixed with 4% paraformaldehyde (PFA) (Nacalai Tesque, Inc., Kyoto, Japan) for 30 min at room temperature (RT) and were then rinsed with PBS twice. Cells were non-permeabilized or permeabilized with 0.5% TritonX-100 in PBS for 20 min and blocked with 3% donkey serum for 1 h at RT. Cells were incubated for 1 h at RT with primary antibodies against a FLAG tag (1:250; Wako, Osaka, Japan), Myc tag (1:250; Wako, Japan), or PDI (1:250; Proteintech, Rosemont, IL, USA) diluted in 1% donkey serum in PBS. Then, the cells were washed three times with PBS, and an Alexa Fluor^®^ 594-conjugated donkey anti-mouse IgG (1:500; Abcam, Cambridge, UK) and Alexa Fluor^®^ 488-conjugated donkey anti-rabbit IgG (1:500; Thermo Scientific Inc.) were added to the cells, which were then incubated for 45 min at RT with shielding. After washing the cells three times with PBS, the cells were mounted with a Vectashield^®^ antifade mounting medium with a DAPI (Vector Laboratories, Burlingame, CA, USA). The images were visualized and captured with a fluorescence microscope (BZ-X700; Keyence, Osaka, Japan) using the appropriate filters.

### 4.4. Flowcytometry

C38 and IB3-1 cells were seeded in a 60 mm culture dish at 6.0 × 10^5^ cells/ dish. After 48 h at a 90% confluence, the cells were harvested by an Accutase^®^ cell detachment solution (Innovative Cell Technologies, Inc., San Diego, CA, USA) and washed with an ice-cold FACS buffer (1 × PBS (pH7.4), 0.1% NaN_3_, 2% FBS). The cells were stained with goat polyclonal anti-SIGIRR (AF990, R&D systems, Minneapolis, MN, USA) or a negative control goat IgG antibody (Jackson ImmunoResearch Laboratories, West Grove, PA, USA) for 30 min on ice. The cells were washed with an ice-cold FACS buffer and stained with AlexaFluor 488^®^ donkey anti-goat IgG secondary antibodies (Thermo Scientific Inc.) for 30 min on ice in the dark. Flow cytometry analysis was performed with an Accuri^TM^ C6 Plus flow cytometer (BD biosciences, San Jose, CA, USA). In each sample, 10,000 cells were analyzed. Collected data were analyzed using FlowJo software (Tree Star, San Carlos, CA, USA).

### 4.5. Constructs and Transfection

Expression plasmids of the WT- and Δ8-SIGIRR were constructed in a pcDNA3.1 (Thermo Scientific Inc.), and the WT and Δ8 SIGIRR tagged with c-Myc or FLAG in the N-terminus were constructed in a pCMV-Myc-N or pCMV-FLAG-N vector (Clontech, Palo Alto, CA, USA), respectively, with standard methods and verified by sequencing. Plasmid DNA was transfected using a FuGene HD transfection reagent (Promega, Madison, WI, USA) according to the manufacturer’s protocols. The FuGene HD/DNA ratios were 3:1.

### 4.6. Immunoblot Analysis

Immunoblot analyses were performed as described previously [54]. The SIGIRR was detected by a goat polyclonal anti-SIGIRR (AF990, R&D systems). Phospho-ERK, -JNK, -p38 MAP Kinase, -IRF-3, ERK, JNK, p38 MAP Kinase, and IκBα were detected by rabbit monoclonal antibodies at 1:1000 or 1:2000 (Cell Signaling Technology, Danvers, MA, USA). β-actin was detected by a mouse monoclonal antibody at 1:20,000 (Sigma-Aldrich, Inc., St. Louis, MO, USA). Calreticulin (CRT) was detected by a mouse monoclonal antibody at 1:5000 (BD biosciences). Sp1 was detected by a rabbit polyclonal antibody at 1:1000 (Active Motif, Carlsbad, CA, USA). FLAG and Myc tags were detected by mouse monoclonal antibodies at 1:1000 (Wako). The antigen-antibody complexes were incubated with an Immobilon Western Chemiluminescent HRP Substrate (Millipore, Burlington, MA, USA) and analyzed by a luminescent image analyzer (LAS-4000mini; FUJIFILM, Tokyo, Japan) to visualize HRP. For reprobing, the membrane was incubated with a 2 M Glycine-HCl (pH2.8) at RT for 1 h. In most of the data in the main text, gels have been cropped for clarity; the bands were confirmed by the comparison with full-length gel images and molecular weight (Supplementary Figure S3).

### 4.7. Biotinylation Assay

After washing the cells three times with cold PBS, the cells were biotinylated by incubation with freshly prepared 1 mg/mL EZ-linkTM Sulfo-NHS-SS biotin (Thermo Scientific Inc.) dissolved in cold PBS (pH8.0) on ice for 1 h, washed five times with cold PBS and solubilized in a RIPA buffer (1% NP-40, 0.1% Sodium Deoxycholate, 150 mM NaCl, 50 mM Tris-HCl, pH7.5). Biotinylated proteins were isolated by incubation with a streptavidin agarose resin (Thermo Scientific Inc.) at 4 °C overnight. After washing six times with a RIPA buffer, biotinylated proteins were eluted with a 4 × SDS sample buffer (Wako).

### 4.8. Enzyme-Linked Immunosorbent Assay (ELISA)

Human IL-8 was measured in a cell culture media by DuoSet^®^ IL-8 ELISA kits (R&D systems) according to the manufacturer’s protocols.

### 4.9. Statistical Analysis

For statistical analysis, the data were analyzed by a Student’s *t*-test or by one-way analysis of variance (ANOVA) with the Tukey–Kramer multiple comparison test (JMP software, SAS Institute, Cary, NC, USA), as indicated in each figure legend. A *p*-value of <0.05 is considered statistically significant.

## Figures and Tables

**Table 1:** Primers used for quantitative and semi-quantitative RT-PCR.

Primer	Orientation	Sequence
Quantitative RT-PCR		
Human WT-SIGIRR	Forward	5′-AGACCCATCTTCATCACCTTCG-3′
	Reverse	5′-GCCAGCTGCACTTCTTTCC-3′
Human Δ8-SIGIRR	Forward	5′-CCTCTTGGTGAACCTGAGCC-3′
	Reverse	5′-ATCGGAGGAAGGAGTCGG-3′
Human 18SrRNA	Forward	5′-GTAACCCGTTGAACCCCATT-3′
	Reverse	5′-CCATCCAATCGGTAGTAGCG-3′
Semi-quantitative RT-PCR		
Human SIGIRR (Exon 7)	Forward	5′-TCTTGGTGAACCTGAGCCG-3′
Human SIGIRR (Exon 9)	Reverse	5′-GCCAGCTGCACTTCTTTCC-3′
Human 18SrRNA	Forward	5′-CGGCTACCACATCCAAGGAA-3′
	Reverse	5′-GCTGGAATTACCGCGGCT-3′

## Data Availability

Not applicable.

## Supplementary Materials

The following are available online at https://www.mdpi.com/article/10.3390/ijms23147748/s1.
