# Supplementary material for: A Splice Switch in SIGIRR Causes a Defect of IL-37-Dependent Anti-Inflammatory Activity in Cystic Fibrosis Airway Epithelial Cells
Source: Int J Mol Sci. 2022 Jul 13;23(14):7748. doi: 10.3390/ijms23147748 (PMC9318995; doi:10.3390/ijms23147748)
Supplement: Supplementary file 1 [file ijms-23-07748-s001.zip › ijms-1742060-supplementary.pdf]

Article

A splice switch in activity in cystic fibrosis airway epithelial cells

Keiko Ueno-Shuto [1], Shunsuke Kamei [2,3,4], Megumi Hayashi [2], Ayami Fukuyama [2], Yuji Uchida [1], Naofumi Tokutomi [1], Mary Ann Suico [2], Hirofumi Kai [2], and Tsuyoshi Shuto [2,*]

SIGIRR causes a defect of IL-37-dependent anti-inflammatory

Supplementary Figures

Figure S1

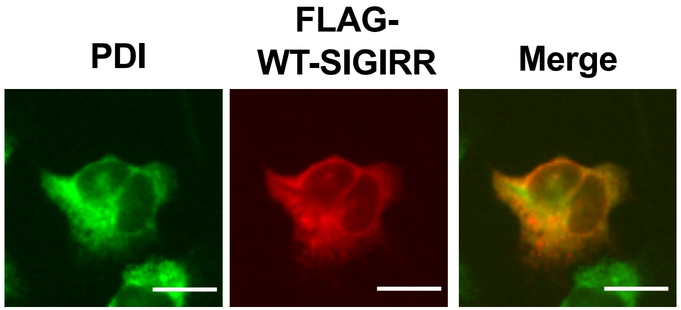

Supplemental Figure S1. Immunofluorescence staining of exogenously expressed Flag-WT-SIGIRR in C38 under permeabilized conditions. Green indicates ER marker protein disulfide isomerase (PDI)-directed Alexa Fluor® 488 and red indicates Flag-directed Alexa Fluor® 594 fluorescence, respectively. Scale bar = 20 μm

Figure S2

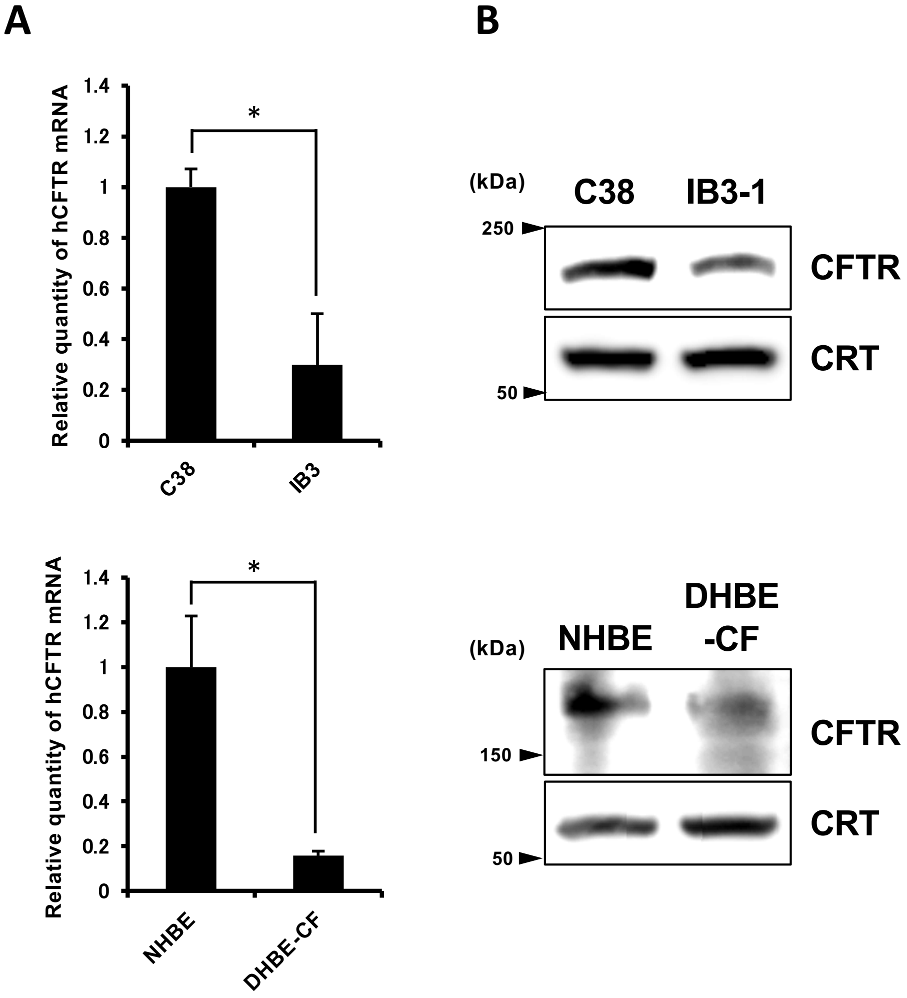

Supplemental Figure S2. (B) The mRNA levels of human CFTR in non-CF and CF cells (C38 and IB3-1, NHBE and DHBE-CF) were assessed by quantitative RT-PCR. The data were normalized to 18SrRNA mRNA levels, as an internal control. *$p<0.05$ versus non-CF cells; Student's t test (n=3). (C) Whole cell lysates in non-CF and CF cells (C38 and IB3-1, NHBE and DHBE-CF) were subjected to immunoblotting using antibody against CFTR. Calreticulin (CRT) was used as a loading control.

Figure S3

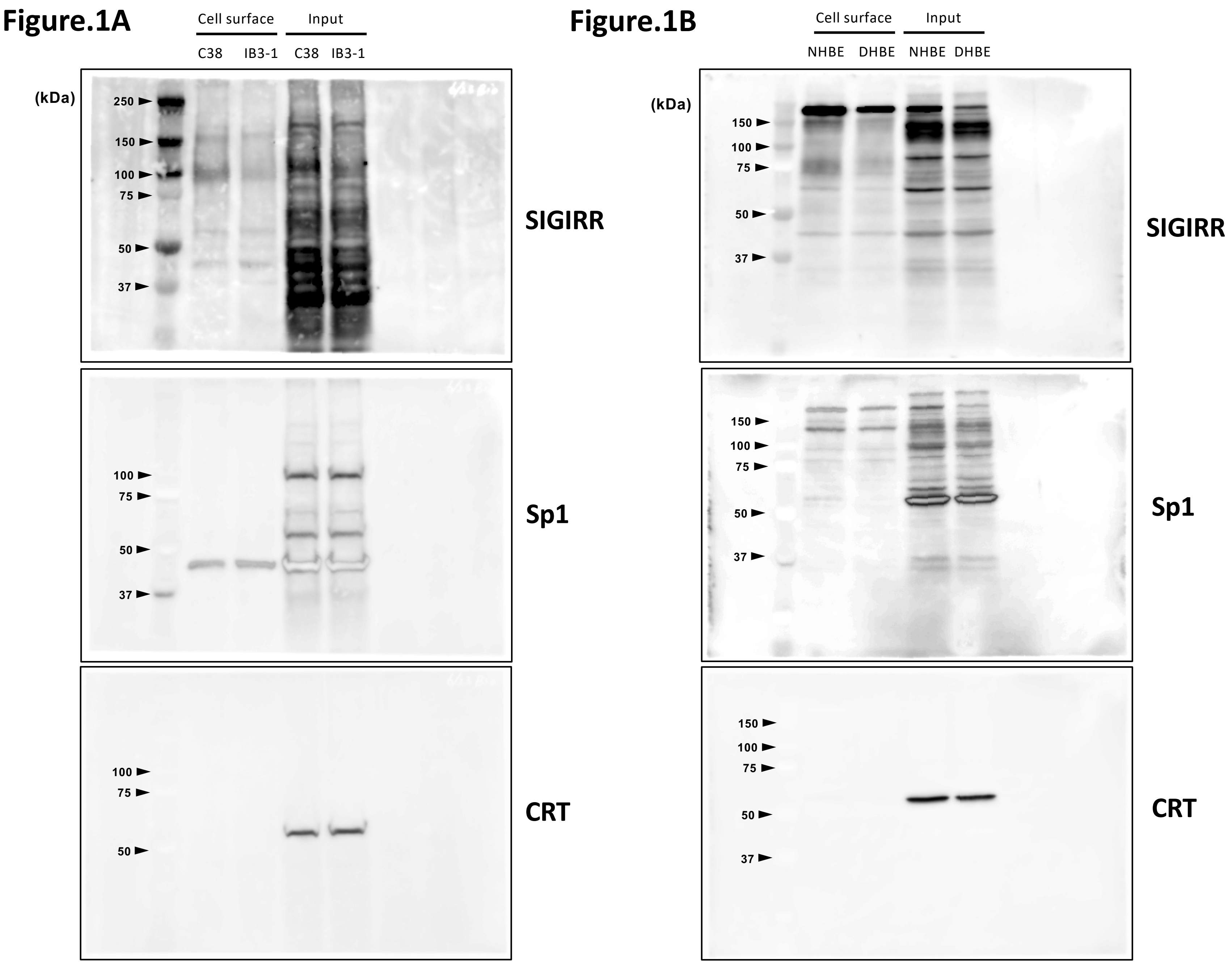

Full-length gel images of Supplementary Figure 1A and 1B.

Figure S3

Figure.2A

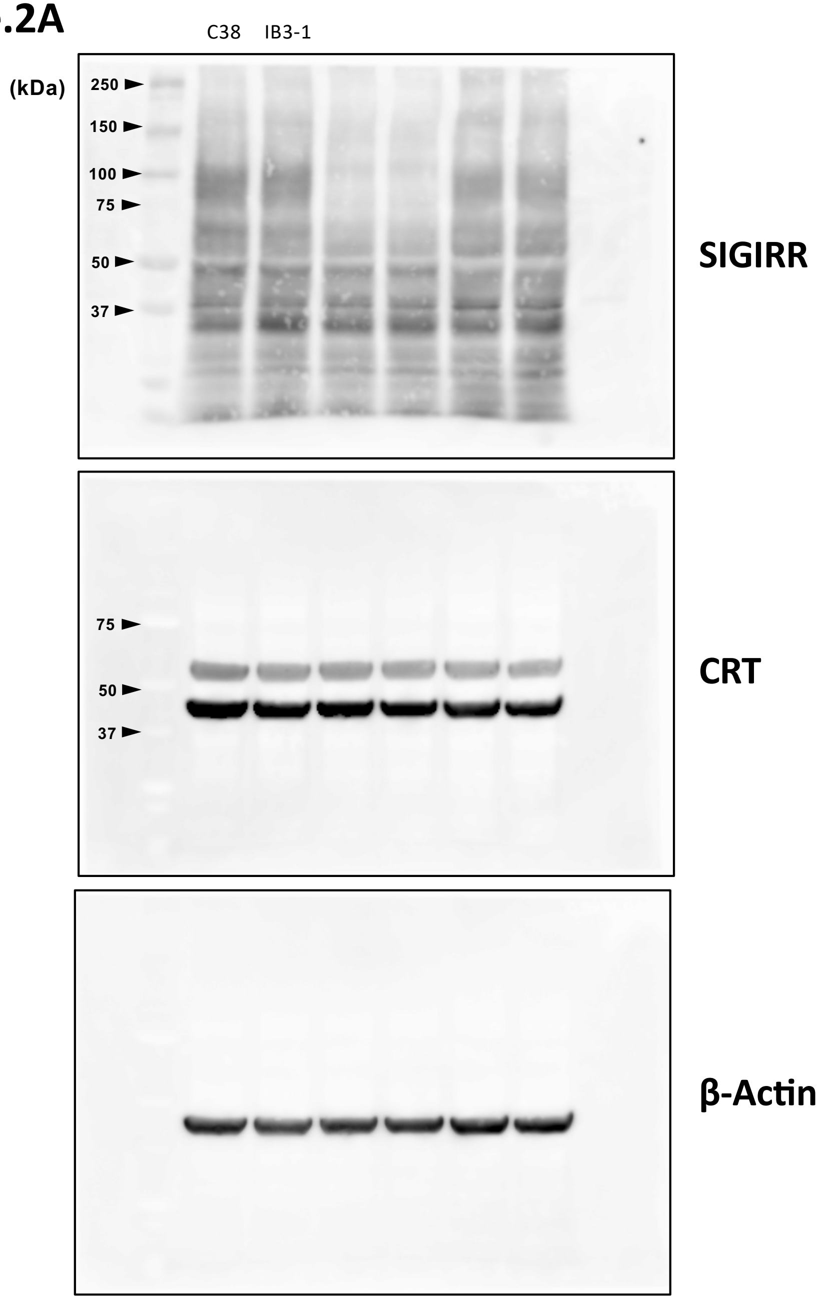

Figure.2C

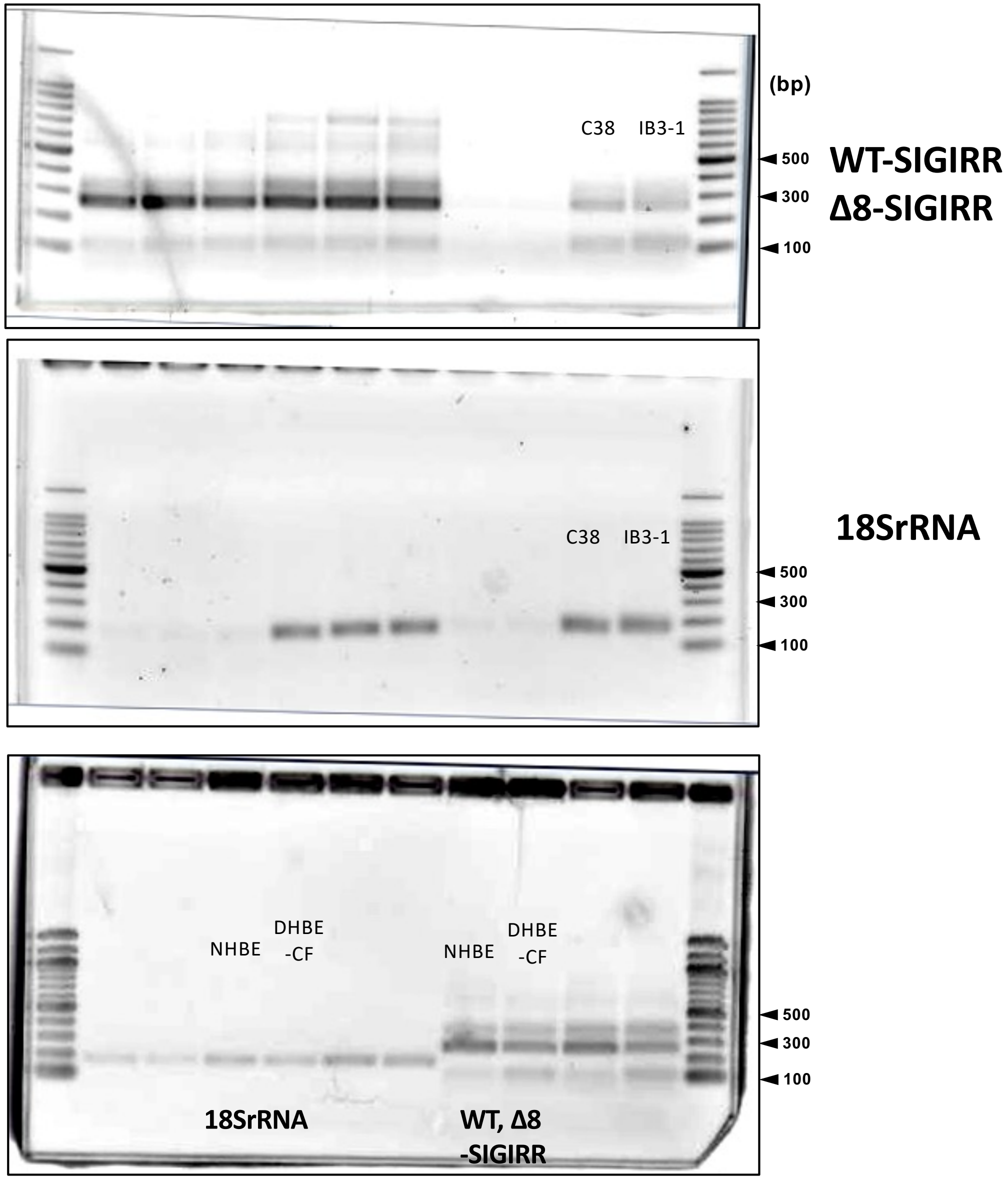

Full-length gel images of Supplementary Figure 2A and 2C.

Figure S3

Figure.2E

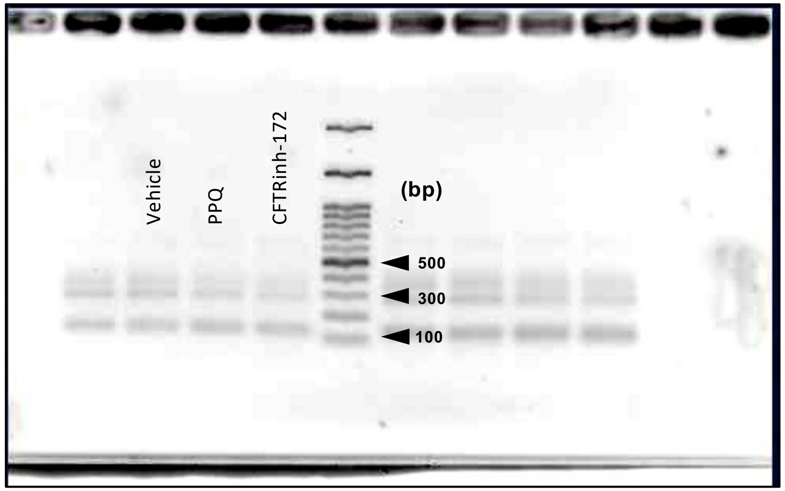

Δ8-SIGIRR

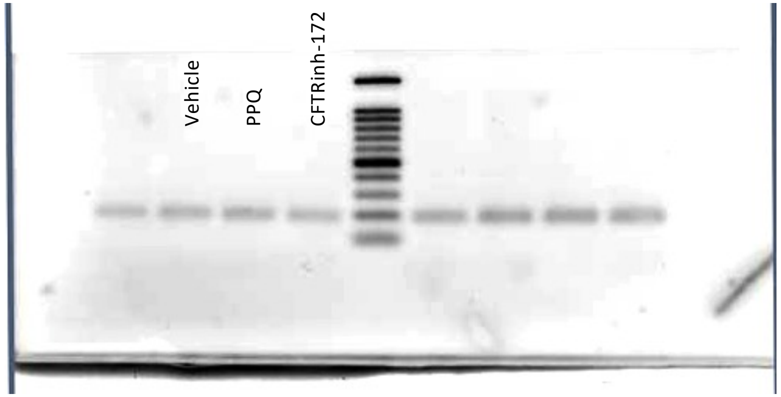

18SrRNA

Full-length gel images of Supplementary Figure 2E.

Figure S3

Full-length gel images of Supplementary Figure 3D.

Figure.3D

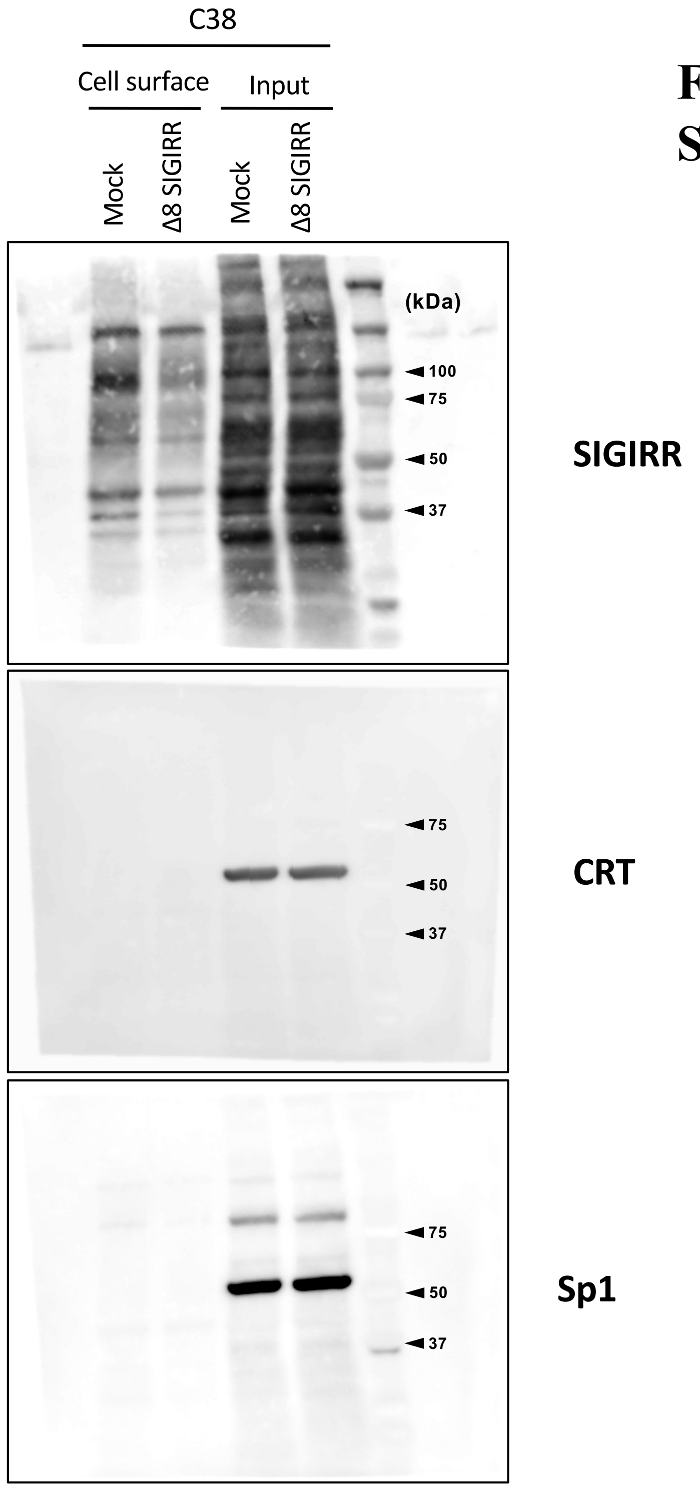

Figure S3

Figure.4D

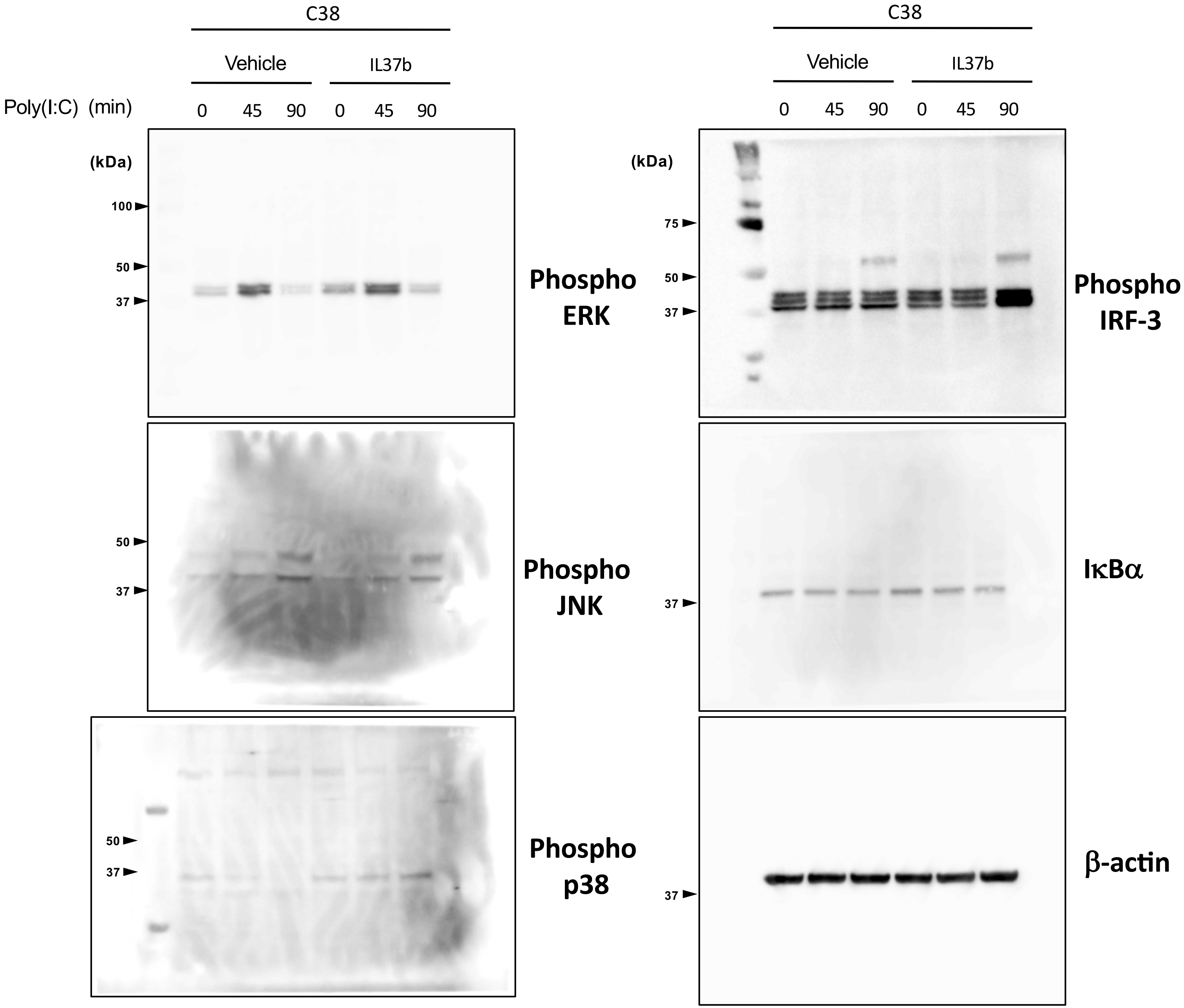

Full-length gel images of Supplementary Figure 4D.

Figure S3

Figure.4D

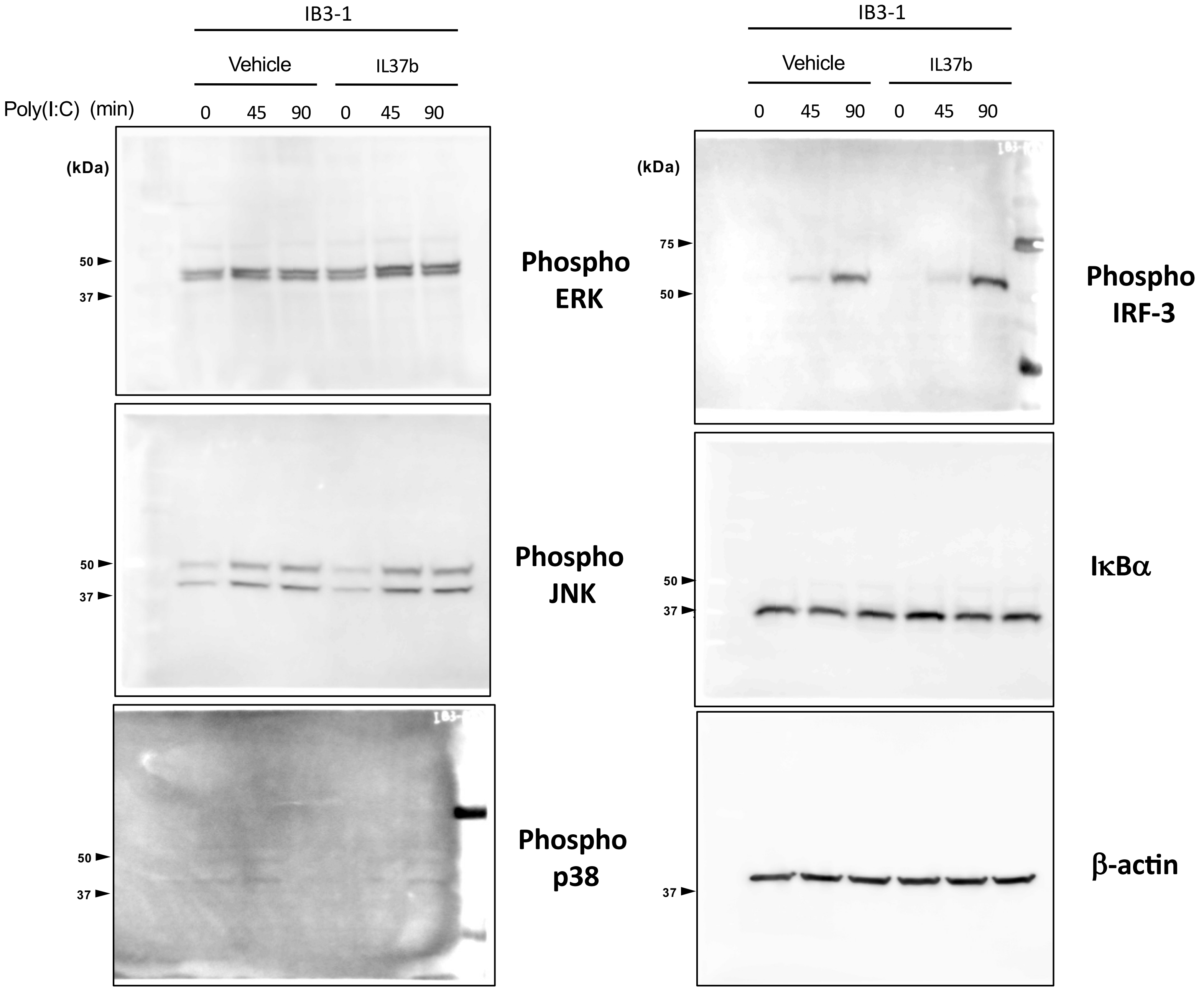

Full-length gel images of Supplementary Figure 4D.

Figure S3

Figure.4E

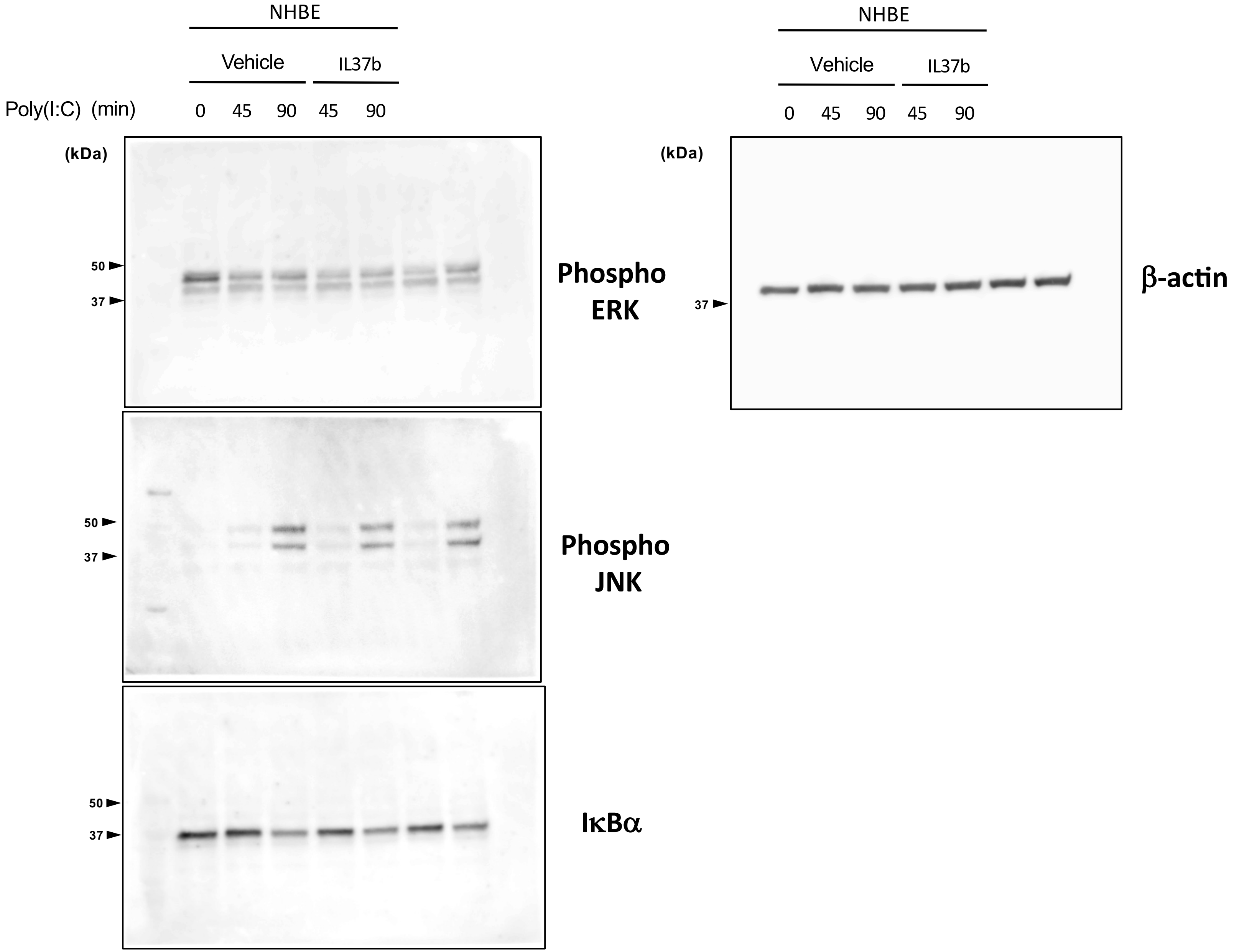

Full-length gel images of Supplementary Figure 4E.

Figure S3

Figure.4E

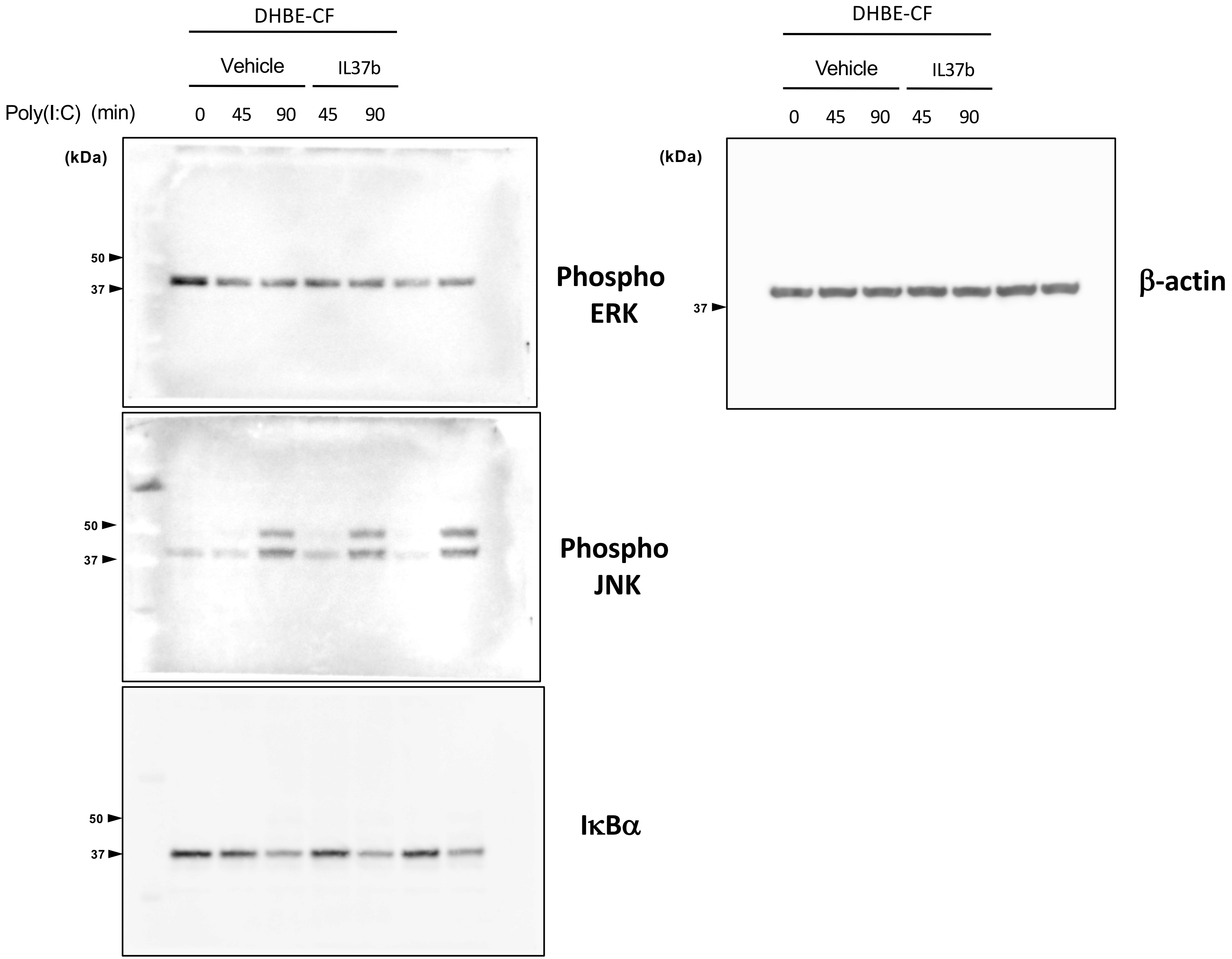

Full-length gel images of Supplementary Figure 4E.

Figure S3

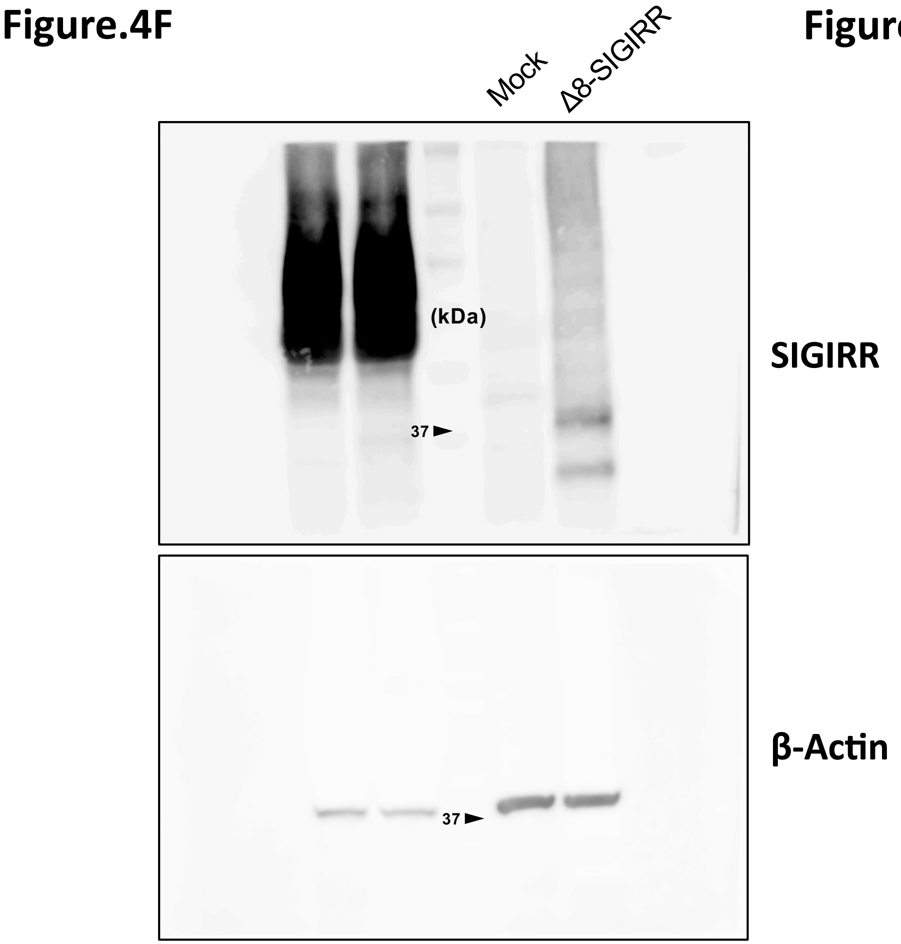

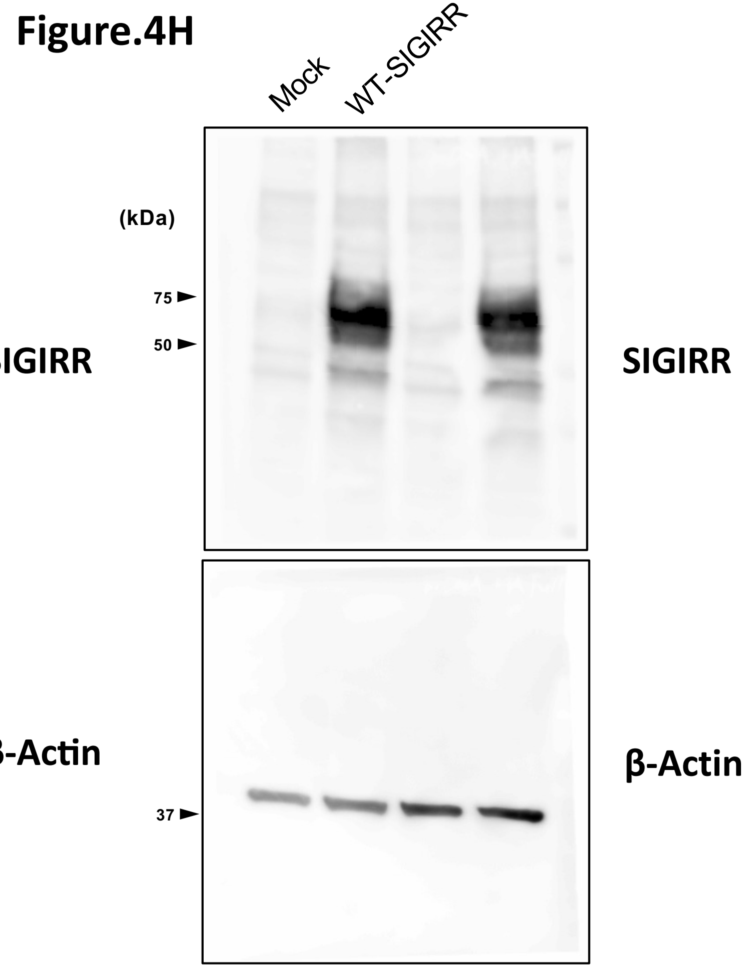

Full-length gel images of Supplementary Figure 4F and 4H.

Figure S3

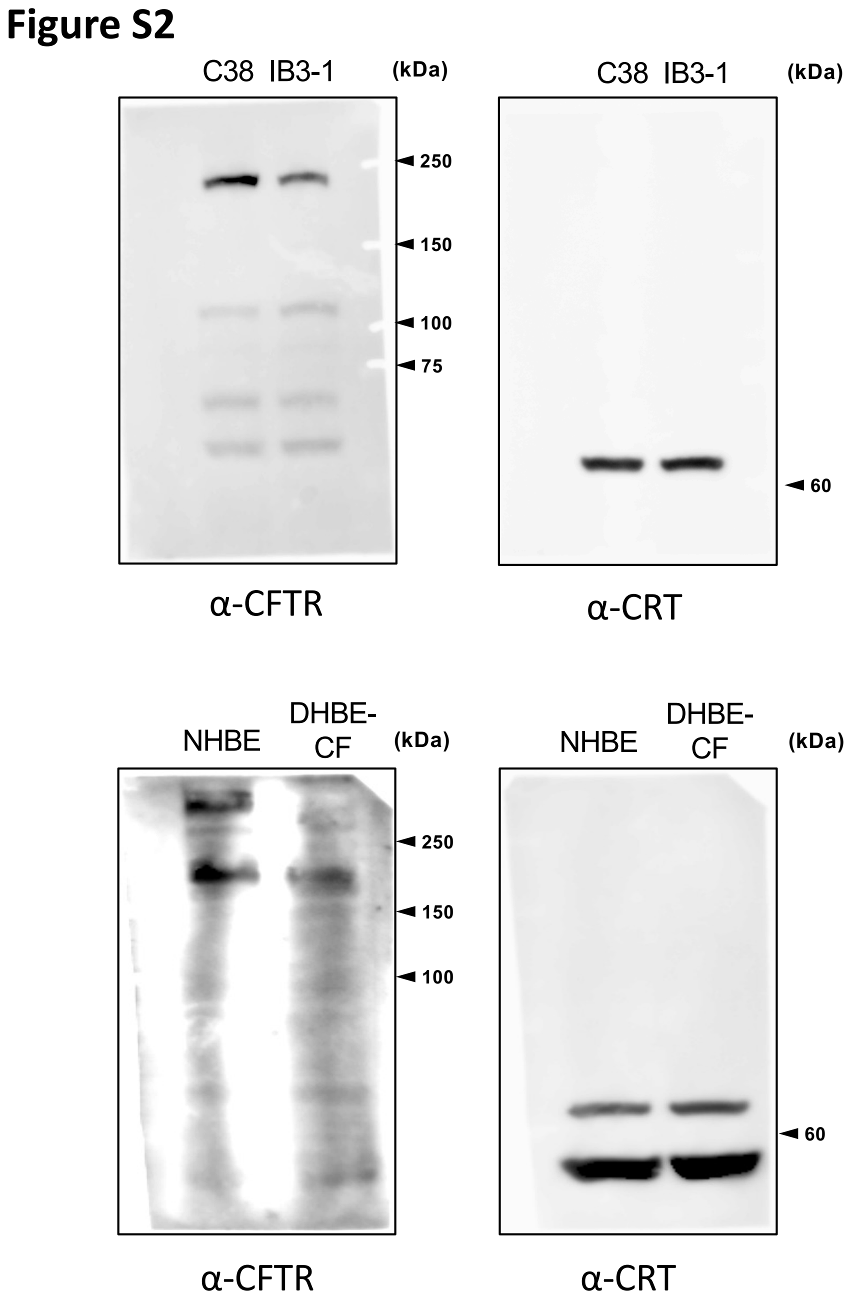

Full-length gel images of Supplementary Figure S2.
